# Comprehensive Analysis of lncRNA-Mediated ceRNA Crosstalk and Identification of Prognostic Biomarkers in Wilms' Tumor

**DOI:** 10.1155/2020/4951692

**Published:** 2020-02-21

**Authors:** Hong Zheng, Bai-Hui Li, Chang Liu, Li Jia, Feng-Ting Liu

**Affiliations:** ^1^Tianjin Medical University Cancer Institute and Hospital, National Clinical Research Center for Cancer, Key Laboratory of Cancer Prevention and Therapy, Tianjin, Tianjin's Clinical Research Center for Cancer, Tianjin 300060, China; ^2^Department of Immunology, Tianjin Medical University Cancer Institute and Hospital, Tianjin 300060, China; ^3^Department of Lung Cancer, Tianjin Medical University Cancer Institute and Hospital, Tianjin 300060, China; ^4^Centre for Haemato-Oncology, Barts Cancer Institute, Queen Mary University of London, London EC1M 6BQ, UK; ^5^Department of Hematology and Oncology, Tianjin Union Medical Center, Tianjin 300191, China

## Abstract

Wilms' tumor (WT) is the most common type of childhood kidney cancer, and most cases present with favorable histology and respond well to standard treatment. However, a subset of patients with WT is diagnosed with bilateral, relapsed, and high-risk tumors which remain the leading cause of cancer-related death in children. Long noncoding RNAs (lncRNAs) and their aberrant expression have currently been attracting great attention as oncogenes or tumor suppressors during tumor initiation and progression. So far, their roles and related competitive endogenous RNA (ceRNA) network remain unelucidated in nephroblastoma pathogenesis. We comprehensively integrated lncRNA, microRNA (miRNA), and messenger RNA (mRNA) expression profiles from the Therapeutically Applicable Research to Generate Effective Treatment (TARGET) database and screened out differentially expressed mRNAs (DEMs), lncRNAs (DELs), and miRNAs (DEMis) to construct a ceRNA network based on the information generated from miRcode, miRTarBase, TargetScan, and miRDB. Gene ontology (GO) and Kyoto Encyclopedia of Genes and Genomes (KEGG) pathway enrichment analyses were performed to analyze the functional characteristics of DEMs in the ceRNA network. The interaction between protein molecules was also analyzed by establishing a protein-protein interaction network. Finally, prognosis-related biomarkers were identified via survival analysis. Initially, 1647 DELs, 115 DEMis, and 3280 DEMs (|log FC| > 2; FDR < 0.01) were obtained using the R package. Next, we constructed a lncRNA-miRNA-mRNA network (ceRNA network), in which 176 DELs, 24 DEMis, and 141 DEMs were identified. Furthermore, 148 functional enrichment terms from GO were identified and 29 KEGG pathways were found to be significantly enriched. We also integrated patient clinical information to analyze the association between DERNAs and patient prognosis. We found that high expression of 8 DELs (LINC00473, AL445228.2, DENND5B−AS1, DLEU2, AC123595.1, AC135178.1, LINC00535, and LMO7−AS1) and 4 DEMs (CEP55, DEPDC1, PHF19, and TRIM36) correlated with poor survival in a patient with WT, whereas high expression of 2 DELs (MEG3 and RMST), 1 DEM (KIAA0922), and 1 DEMi (hsa−mir−200a) could possibly lead to better clinical outcomes. For the first time, the present study provided a novel insight into lncRNA-related ceRNA networks and identified potential prognostic biomarkers in Wilms' tumor.

## 1. Introduction

Wilms' tumor (WT), also known as nephroblastoma, is an embryonal childhood tumor that accounts for 90% of childhood renal tumors and 7% of all childhood cancers [1]. Due to the successive clinical trials conducted by several collaborative clinical trial groups around the world, such as the International Society of Paediatric Oncology (SIOP), and the National Wilms' Tumor Study Group (NWTSG), treatment regimen that the combination of nephrectomy and chemotherapy was found to greatly improve overall survival (OS) rates exceeding 90% [2]. Overall, WT treatment can be considered as a successful story. However, the survival rates of a subgroup of patients with WT, which include those with poor histological and molecular features, bilateral disease, and recurrent disease, are still well below 90% [3]. Therefore, investigators continue to look for the molecular pathogenesis of WT with the belief that novel treatment strategies will be conducive to improving outcomes in patients with high-risk tumors. Notably, novel biomarkers are expected to predict the prognostic outcomes in patients with WT.

MicroRNAs (miRNAs) are ∼22-nucleotide (nt) noncoding RNAs that negatively regulate gene expression at the posttranscriptional levels [4]. Accumulated evidences show that the aberrant expression of miRNAs, such as miR-17∼92 cluster, miR-185, miR-204, and miR-483, play important roles in WT pathogenesis [5]. Long noncoding RNAs (IncRNAs) are defined as RNA transcripts that are longer than 200 nt with no potential of protein coding; however, they exhibit greater tissue specificity than miRNAs and messenger RNAs (mRNAs) [6]. It was reported that lncRNAs play an important role in regulating tumor progression and tumor biological behaviors such as gene expression, cell proliferation, cell differentiation, immune responses, and apoptosis, with the most crucial process being the regulation of competing endogenous RNAs (ceRNAs) [7, 8]. The ceRNA hypothesis, first proposed by Salmena et al. in 2011, gained a lot of attention in terms of tumorigenesis [9]. Basically, the hypothesis states that, in the ceRNA gene interaction network, which consists of lncRNAs, miRNAs, and mRNAs, lncRNAs can act as miRNA sponges and inhibit miRNA functions by sharing miRNA response elements (MRE), thereby indirectly regulating mRNA expression levels [10]. Hence, the idea that lncRNAs could serve as promising biomarkers for multiple diseases has gained increasing attention. Currently, the importance of the lncRNA-miRNA-mRNA ceRNA regulatory network has been confirmed in various types of cancers [11–13]. However, only few studies on the roles of lncRNAs have been conducted in WT. Moreover, lncRNA-related ceRNA network remains unelucidated in WT.

In this study, we used RNA sequencing data from the TARGET Data Matrix database to identify differentially expressed lncRNAs, miRNAs, and mRNAs. Subsequently, we performed a series of analyses, including ceRNA network construction, functional enrichment analyses, protein-protein interaction (PPI) analyses, and survival analyses. Thus, the present study aimed to identify the ceRNA regulatory mechanism involved in WT pathogenesis and provide potential biomarkers for WT prognosis, thereby improving the survival rate of patients with high-risk WT.

## 2. Materials and Methods

### 2.1. Study Population and RNA-Sequencing Data Processing

Two expression profiles were downloaded for subsequent analysis from TARGET Data Matrix (https://ocg.cancer.gov/programs/target/data-matrix), one for miRNA expression profile and the other one contained mRNA and lncRNA expression profile. miRNA expression profile data were acquired from 132 WT and 6 normal tissues. mRNA and lncRNA expression profile data were acquired from 126 WT and 6 normal tissues. Then, we used parametric extraction language to extract mRNA and lncRNA expression profile data separately. The R software package was used to process the downloaded files and to convert and reject the unqualified data. The data were calibrated, standardized, and log 2 transformed. TARGET investigators analyzed tumors from patients who relapsed and for whom standard therapies proved ineffective to identify biomarkers that correlated with poor clinical outcome and/or new therapeutic approaches. The tissues used in this study were collected from patients enrolled in the National Wilms' Tumor Study (mostly NWTS-5) clinical trial, which was completed by the Children's Oncology Group (COG).

### 2.2. Identification of DELs, DEMis, and DEMs

The differential expression of mRNAs, lncRNAs, and miRNAs between WT and normal tissues were analyzed using the edgeR, an R package that processes the count data based on the negative binomial distribution model to analyze gene differential expression. [14]. First, the “Trimmed Mean of M-values” (TMM) normalization method was used to calibrate the reading length of the gene, and then the negative binomial distribution model was used to calculate the differential expression *P* values of the RNAs. Next, the FDR was used to modify the *P* value. The screening conditions for mRNA, miRNA, and lncRNA differential expression were |log FC| > 2, FDR < 0.01. Finally, volcano plots were generated for the DELs, DEMis, and DEMs that were obtained using ggplot2 package in the R software.

### 2.3. Construction of the ceRNA Network

The ceRNA network construction included the following steps: (1) DEL-DEMi interactions were predicted using miRcode (http://www.mircode.org/) [15]; (2) by comparing the data, the mutual regulation relationship between DEL-DEMi was determined and screened out; (3) the target genes of DEMis selected from the previous step were predicted using the miRDB (http://www.mirdb.org/miRDB/) [16], miRTarBase (http://mirtarbase.mbc.nctu.edu.tw/) [17], and TargetScan databases (http://www.targetscan.org/) [18], and only the target genes simultaneously recognized by these three databases were considered as candidate target genes; (4) the overlapping genes were obtained by intersecting DEMs and the target genes; (5) the lncRNA-miRNA-mRNA ceRNA network was then constructed and visualized using Cytoscape v3.7.1 [19]; and (6) the ceRNA subnetwork was generated using the Molecular Complex Detection (MCODE) plug-in Cytoscape.

### 2.4. Functional Enrichment Analysis

Gene Ontology (GO) and Kyoto Encyclopedia of Genes and Genomes (KEGG) pathway enrichment analyses of the code genes involved in the ceRNA network were performed using clusterProfiler v3.6.0, which is an ontology-based R package [20]. A *P* value < 0.05 was considered as statistically significant.

### 2.5. PPI Network Construction and Module Analysis

The Search Tool for the Retrieval of Interacting Genes (STRING; http://string-db.org) online database was used to construct the DEM PPI network within the ceRNA network [21]. Combined score >0.4 was considered to be statistically significant. Next, the data from STRING were obtained to model the PPI network using Cytoscape v3.7.1. Densely connected regions in the PPI network were identified using MCODE.

### 2.6. Survival Analysis

In this study, the clinicopathological data and differential gene expression data of patients with WT in the TARGET Data Matrix were combined, and the overall survival curve of each differentially expressed RNA was generated using the “survival” package (version 2.41.3; https://cran.r-project.org/web/packages/survival/index.html) in R. Due to lack of clinical information of some patients, clinical data of 120 WT patients were finally collected. The clinical features of WT patients include age, gender, race, and TNM stage. SPSS was used for univariate and multivariate analysis to evaluate the impact of clinical features on OS of 120 patients. All data were processed using logarithmic transformation and median centralization. The Kaplan–Meier one-way survival method was used for analysis. A *P* value < 0.05 was considered as statistically significant.

## 3. Results

### 3.1. Identification of DEMs, DEMis, and DELs

**Figure 1:**
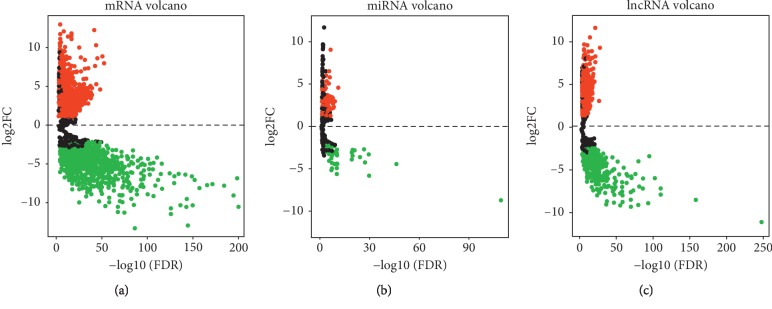
Volcano plots of differentially expressed RNAs. (a) Volcano plots of differentially expressed mRNAs (DEMs). The red dot represents upregulated DEMs, and green dot represents downregulated DEMs. (b) Volcano plots of differentially expressed miRNAs (DEMis). The red dot represents upregulated DEMis, and green dot represents downregulated DEMis. (c) Volcano plots of differentially expressed lncRNAs (DELs).

A total of 3280 DEMs, 115 DEMis, and 1647 DELs were confirmed using the “edgeR” package in R. Notably, 1580 DEMs out of 3280 were upregulated, and the remaining 1700 were downregulated (Figure 1(a), Table 1). There were 68 upregulated and 47 downregulated DEMis (Figure 1(b), Table 2). Finally, out of 1647 DELs, 875 were upregulated and 772 were downregulated (Figure 1(c), Table 3).

### 3.2. Construction of the ceRNA Network in Wilms' Tumor

**Figure 2:**
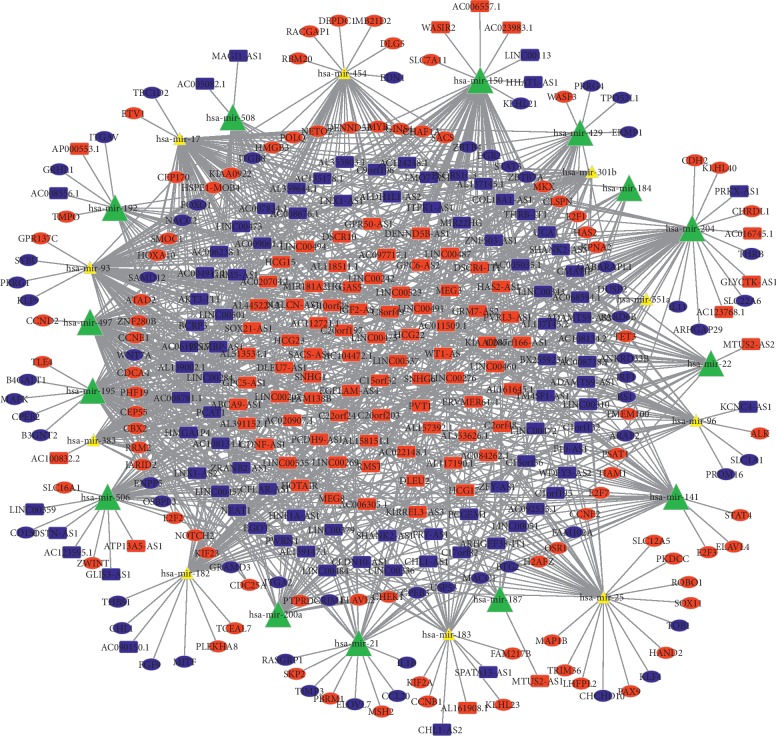
CeRNA network in WT. Red dots indicate upregulated mRNAs, blue dots indicate downregulated mRNAs, yellow triangles represent upregulated miRNAs, green triangles represent downregulated miRNAs, red rectangles indicate increased expression of lncRNAs, and blue rectangles indicate decreased expression of lncRNAs.

**Figure 3:**
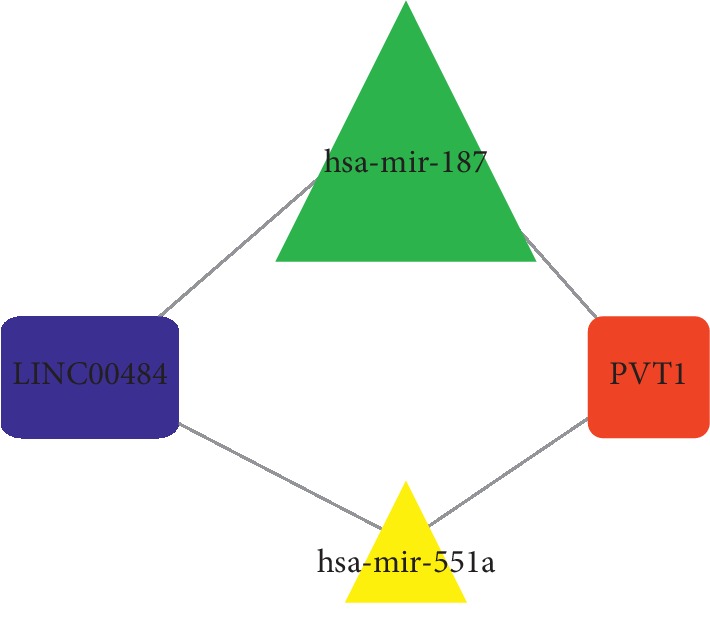
Densely connected region of ceRNA network. The red rectangle indicates increased expression of lncRNA, blue rectangle indicates decreased expression of lncRNA, green triangle represents downregulated miRNA, and yellow triangle represents upregulated miRNA.

Based on the miRcode database, 800 pairs of interacting lncRNAs and miRNAs were identified, consisting of 24 DEMis and 176 DELs (Table 4). Next, we used TargetScan, miRDB, and miRTarBase databases to predict interactions between miRNAs and mRNAs, and 1193 target genes simultaneously recognized by these three databases were considered as the target genes of 24 DEMis (Table 5). These target genes then intersected with 3280 DEMs, and we identified 141 mRNAs from the 3280 DEMs. Finally, we constructed the lncRNA-miRNA-mRNA ceRNA network according to DEL-DEMi interactions and the DEMi-DEM interactions using Cytoscape and constructed the lncRNA-miRNA-mRNA ceRNA network using Cytoscape. In the ceRNA network, 176 DELs, 24 DEMis, and 141 DEMs were identified (Figure 2). Out of 141 DEMs, 81 were upregulated and 60 were downregulated. Using MCODE in Cytoscape, 4 key genes (PVT1, hsa-mir-187, hsa-mir-551a, and LINC00484), which may play an important role in the development of nephroblastoma, were found to be densely connected (Figure 3).

### 3.3. GO and KEGG Pathway Analysis of the 141 DEMs Involved in the ceRNA Network

**Figure 4:**
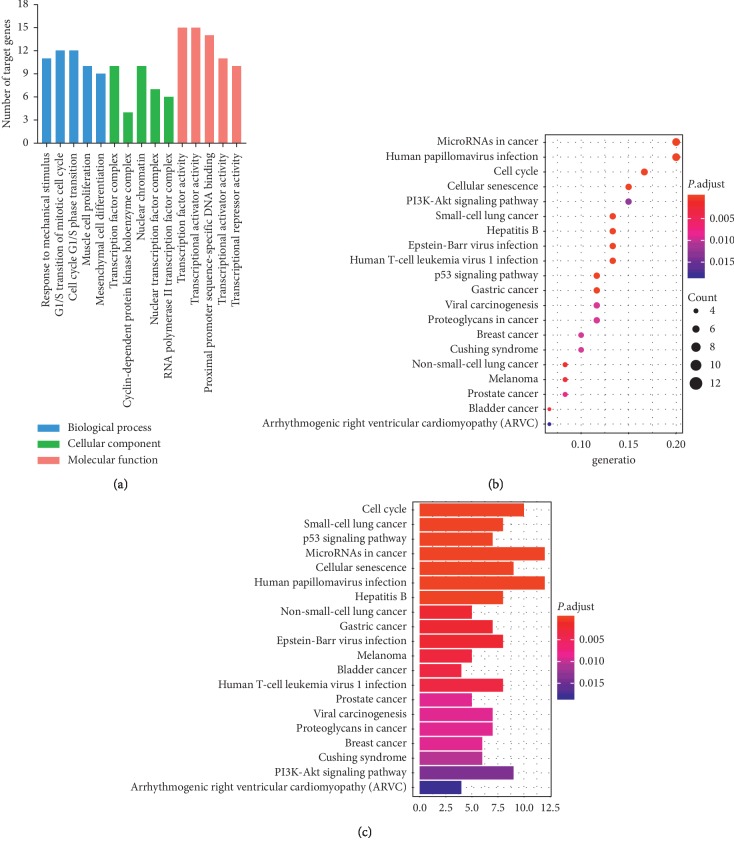
GO and KEGG functional enrichment analysis of target genes involved in the ceRNA regulation network of WT. (a) GO functional enrichment analysis. The bar graphs show GO functional enrichment analysis with horizontal axis for GO items and vertical axis for number of target genes enriched in GO terms. The color blue represents the biological process, green represents the cellular component, and red represents the molecule function. (b, c) KEGG functional enrichment analysis. The bar and bubble plots show KEGG pathway enrichment data.

To further analyze the functional characteristics of the 141 DEMs in the ceRNA network, we used the clusterProfiler package to perform GO and KEGG pathway analysis. Overall, 148 functional enrichment terms from GO were observed to be divided into 3 parts, including 131 biological processes (BPs), 8 cellular components (CCs), and 9 molecular functions (MFs). The top 5 BPs included “response to mechanical stimulus,” “G1/S transition of mitotic cell cycle,” “cell cycle G1/S phase transition,” “muscle cell proliferation,” and “mesenchymal cell differentiation.” The top 5 CCs included “transcription factor complex,” “cyclin-dependent protein kinase holoenzyme complex,” “nuclear chromatin,” “nuclear transcription factor complex,” and “RNA polymerase II transcription factor complex.” The top 5 MFs included “transcription factor activity, RNA polymerase II proximal promoter sequence-specific DNA binding,” “transcriptional activator activity, RNA polymerase II transcription regulatory region sequence-specific DNA binding,” “proximal promoter sequence-specific DNA binding,” “transcriptional activator activity, RNA polymerase II proximal promoter sequence-specific DNA binding,” and “transcriptional repressor activity, RNA polymerase II transcription regulatory region sequence-specific DNA binding” (Figure 4(a)). In our analysis, 29 KEGG pathways were also observed to be significantly enriched. Major pathways associated with Wilms' tumor included “Cell cycle,” “p53 signaling pathway,” “MicroRNAs in cancer,” and “PI3K-Akt signaling pathway” (Figures 4(b) and 4(c)).

### 3.4. PPI Network Construction of the 141 DEMs Involved in the ceRNA Network

**Figure 5:**
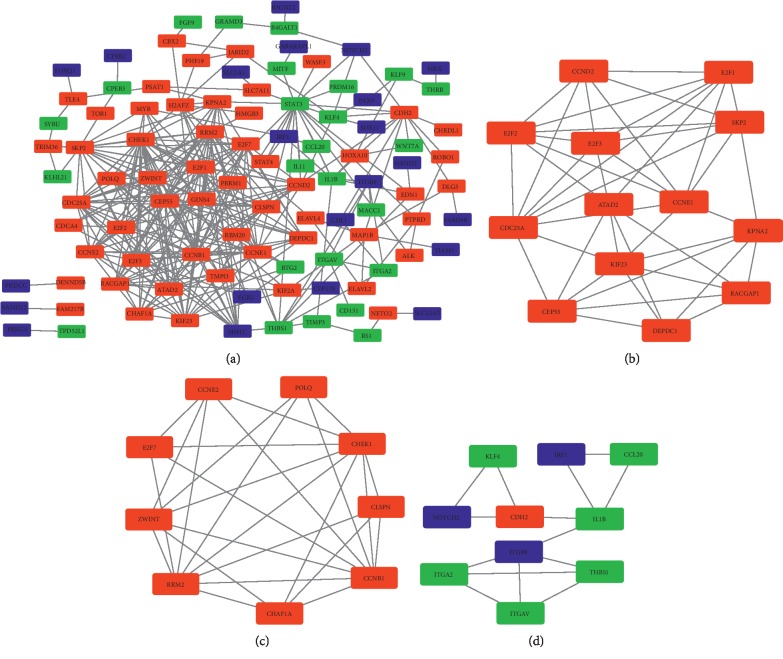
Protein-protein interaction network of target genes in the ceRNA network. (a) PPI network in WT. The red rectangles represent upregulated DEMs, green rectangle represent downregulated DEMs, and blue rectangles represent DEMs that are not in the ceRNA but are associated with the target genes. (b–d) Densely connected regions of the PPI network. The red rectangles represent upregulated DEMs, green rectangle represent downregulated DEMs, and blue rectangles represent DEMs that are not in the ceRNA but are associated with the target genes.

The PPI network of the 141 DEMs involved in ceRNA network was constructed (Figure 5(a)). MCODE identified 3 significant modules in the PPI network. The first module consisted of 13 target genes, including KPNA2, DEPDC1, RACGAP1, SKP2, CCNE1, E2F2, CEP55, CCND2, E2F1, ATAD2, E2F3, CDC25A, and KIF23 (Figure 5(b)). The second module consisted of 9 target genes, including CHEK1, POLQ, E2FT, ZWINT, CCNB1, CCNE2, RRM2, CHAF1A, and CLSPN (Figure 5(c)). The third module consisted of 10 target genes, including IRF1, NOTCH2, IL1B, KLF4, CCL20, ITGB8, THBS1, CDH2, ITGA2, and ITGAV (Figure 5(d)).

### 3.5. Survival Analysis

**Figure 6:**
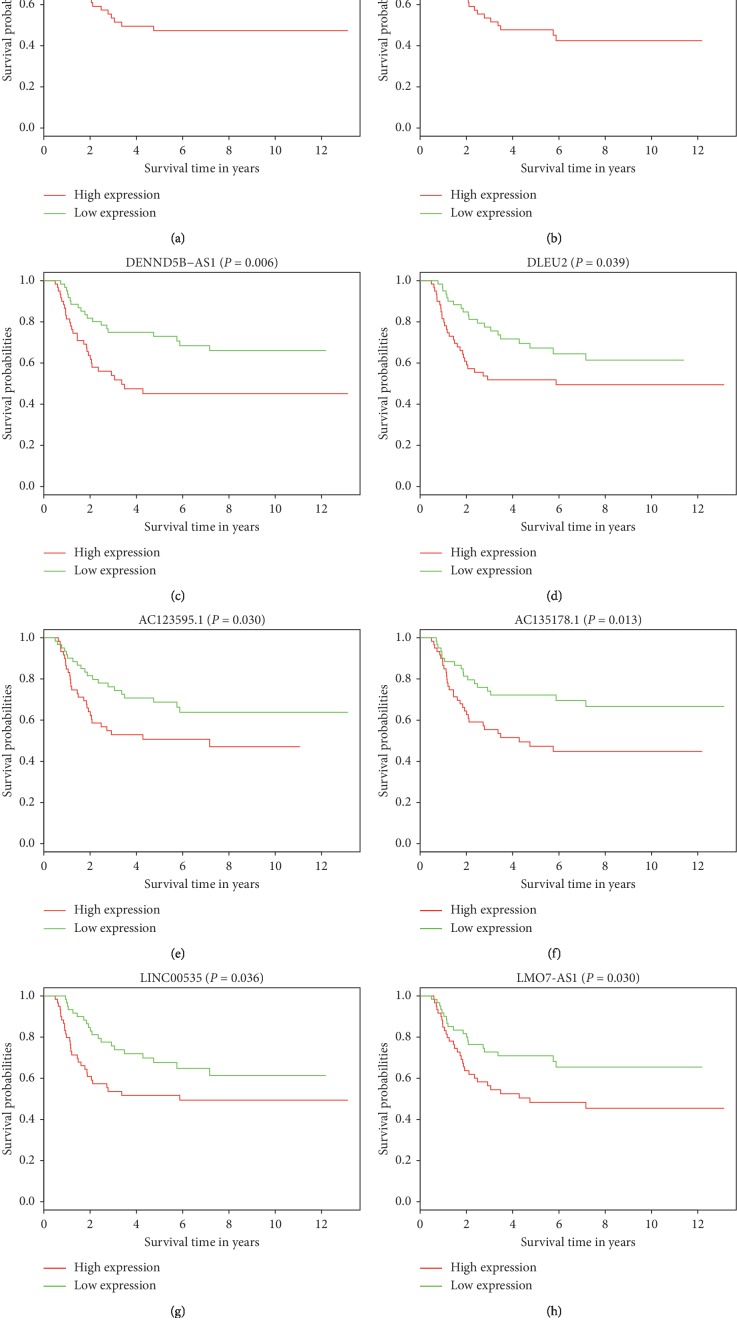
Survival analysis for DELs, DEMs, and DEMis in WT. (a–h) Survival analysis for 8 differentially expressed RNAs in WT. *X*-axis and *Y*-axis represent survival time in years and survival probability.

**Figure 7:**
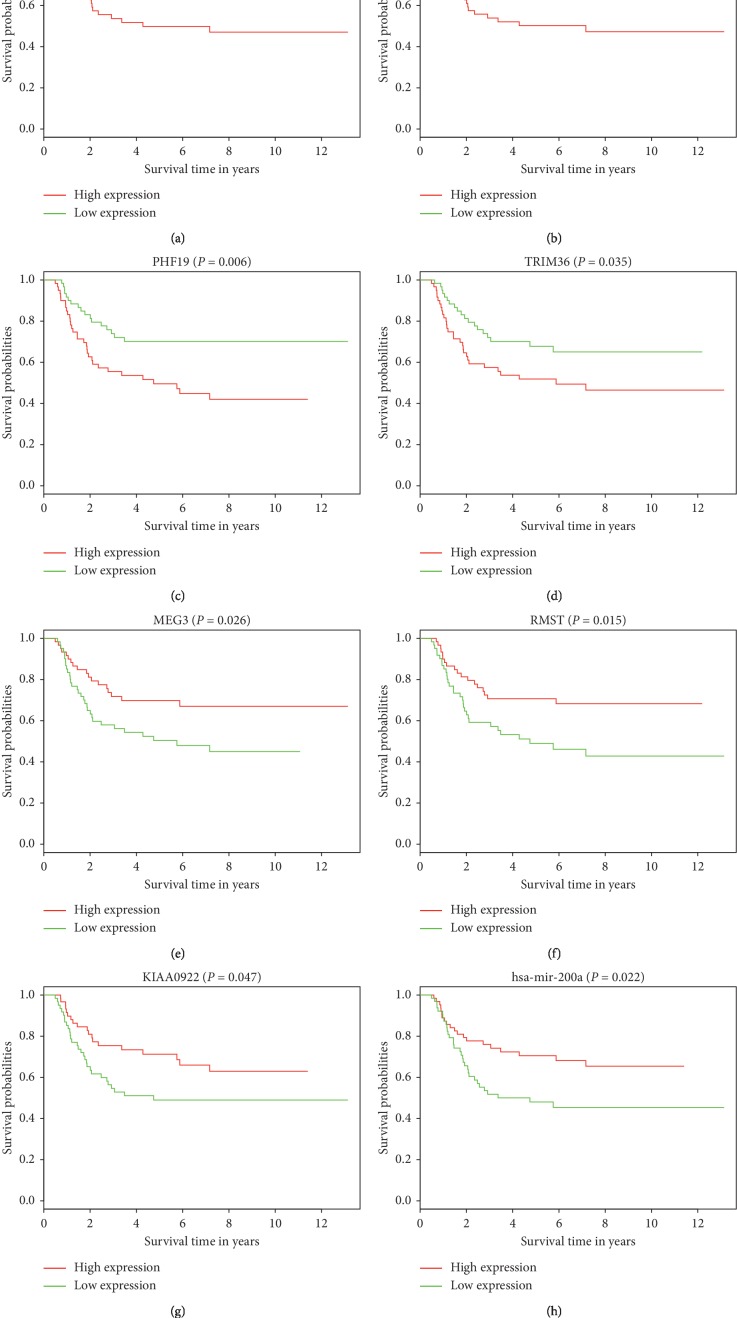
Survival analysis for DELs, DEMs, and DEMis in WT. (a–h) survival analysis for 8 differentially expressed RNAs in WT. *X*-axis and *Y*-axis represent survival time in years and survival probability.

The clinical feature of WT patients is shown in Table 6. Results of the univariate and multivariate analysis are shown in Table 7. The gender (*P*=0.032) and TNM stage (*P*=0.015) were significantly associated with OS in univariate analysis. Then, we selected these two variables in multivariate analysis. In multivariate analysis, we found that gender (HR = 0.432, 95% CI: 0.242 to 0.773, *P* = 0.005) and TNM stage (HR = 1.674, 95% CI: 1.203 to 2.329, *P*=0.002) were independent prognostic factors for OS. Out of 176 DELs, 10 were associated with prognosis in patients with WT. Among these 10 DELs, high expression of 8 DELs (LINC00473, AL445228.2, DENND5B−AS1, DLEU2, AC123595.1, AC135178.1, LINC00535, and LMO7−AS1) was associated with poor survival (Figures 6(a)–6(h)). Similarly, high expression of 4 DEMs (CEP55, DEPDC1, PHF19, and TRIM36) was found to exhibit poor prognosis (Figures 7(a)–7(d)). Conversely, we found that high expression of 2 DELs (MEG3, RMST), 1 DEM (KIAA0922), and 1 DEMi (hsa−mir−200a) correlated with improved survival (Figures 7(e)–7(h)).

## 4. Discussion

With the development of high-throughput sequencing technology and bioinformatics, ncRNA dysregulation has been found to be related to tumorigenesis, neurological disorders, cardiovascular diseases, and developmental diseases among others [22]. The ceRNA hypothesis particularly provides a new perspective in terms of studying tumor formation mechanisms and the developing cancer treatments. In this study, we first screened significantly altered IncRNAs, miRNAs, and mRNAs in patients with WT from the TARGET database. Next, 176 DELs, 24 DEMis, and 141 DEMs were used to construct a ceRNA modulation network. Cluster analysis of the ceRNA network revealed 4 key genes, which consisted of PVT1, hsa-mir-187, hsa-mir-551a, and LINC00484. While PVT1, hsa-mir-187, and hsa-mir-551a were found to be upregulated, LINC00484 was found to be downregulated. GO and KEGG pathway analysis demonstrated that “cell cycle” and “p53 signaling pathway” probably played an important role in WT recurrence. Furthermore, the p53 transcription factor not only observed to induce protein coding genes, but also regulated noncoding RNA, including PVT1, lncRNA-p21, and miR-34a [23]. PVT1, a large (>300 kb) locus on the human chromosome 8q24 adjacent to the c-myc locus, is an oncogene that is involved in cell proliferation, lymph node infiltration, apoptosis, and metastasis [7, 23, 24]. Notably, its overexpression in cancers, such as non-small-cell lung cancer, prostate cancer, acute promyelocytic leukemia, and hepatocellular carcinoma, has been confirmed [24–27]. However, no reports exist between PVT1 and WT. Additionally, the relationship between PVT1, the 2 miRNAs (has-mir-187 ahashsa-mir-551a), and LINC00484 has also not been reported. We speculate that PVT1 interacts with P53 to promote tumor progression by regulating cell cycle in WT. Therefore, studying the mechanisms that regulate PVT1 expression may result in therapeutic benefits for patients with WT.

Furthermore, we constructed the PPI network and performed survival analysis. A total of 32 crucial DEMs were identified, and several molecules were screened out to correlate with the prognosis of patients with WT. Among these DEmRNAs, we found that high DEPDC1 and CEP55 expression was associated with poor prognosis in patients with WT based on our survival analysis results. Several studies showed that the DEP domain containing 1 (DEPDC1) protein was a novel oncoantigen that was aberrantly overexpressed in multiple types of cancers [28, 29]. DEPDC1 was shown to be negatively regulated by miR-26b and promoted cell proliferation and tumor growth by upregulating FOXM1 expression in triple-negative breast cancer [30]. CEP55 (centrosome protein 55KDa) is a key regulator of cytokinesis via Erk2/Cdk1-dependent phosphorylation at S425 and S428 [31]. Recently, several studies showed that CEP55 regulated cancer progression. Kalimutho et al. reported that CEP55 was a downstream effector of the MEK 1/2-MYC axis and that blocking this pathway was beneficial while treating breast cancer [32]. High CEP55 expression was also closely related to poor prognosis in patients with liver cancer, as it promoted the migration and invasion of liver cancer cells by regulating JAK2-STAT3-MMP signaling [33]. Furthermore, CEP55 was shown to activate CCND1 and FN1 via the AKT signaling pathway, thereby regulating the proliferation and invasion of osteosarcoma cells [33]. However, the relationship between DEPDC1 or CEP55 and WT still needs to be determined. Thus, we hope to bring new perspectives to all the relevant researchers involved in this subject.

In this study, 10 DELs were identified to be associated with prognosis. This indicated that lncRNA may play a role in the progression of WT. Hopefully, this can form a basis for studies conducted to determine the role of lncRNA in WT development. The results demonstrated that high expression of LINC00473, which sponged hsa-mir-150, hsa-mir-195, and hsa-mir-497 to indirectly regulate target genes, was associated with poor prognosis. LINC00473 was already confirmed to be involved in the molecular pathogenesis of WTs via miR-195/IKKα, and high LINC00473 levels were associated with higher stage and adverse histology WT [34]. According to our analysis, CEP55 was one of the target genes that were regulated by hsa-mir-195. Thus, we hypothesized that abnormal upregulation of LINC00473 sponged hsa-mir-195, which inhibited its negative regulation of CEP55, thereby upregulating CEP55 and resulting in WT. Unfortunately, we lack relevant conditions to verify this speculation using experiments. Contrastingly, we also found that high expression of maternally expressed gene 3 (MEG3) led to improved prognosis. A previous study showed that MEG3 could inhibit tumorigenesis in a p53-dependent manner [35]. However, the role of MEG3 in WT suppression requires experimental verification. As for the remaining 8 DELs, they still lack relevant reports unfortunately.

## 5. Conclusions

In summary, for the first time, this study identified a ceRNA network that regulated WT progression and predicted the crucial genes and pathways associated with WT. Thus, these findings may provide potential biomarkers for the diagnosis and prognosis of high-risk nephroblastoma.

## Figures and Tables

**Table 1:** The top 10 differentially expressed mRNAs.

mRNA	log FC	log CPM	*P* value	FDR
Upregulated				
SOX11	8.24369	8.12664	1.31*E* − 52	1.18*E* − 50
EYA1	9.12925	8.5296	8.96*E* − 51	7.51*E* − 49
NUSAP1	4.86672	7.60798	3.05*E* − 48	2.27*E* − 46
SIX1	8.88707	6.71694	4.02*E* − 45	2.77*E* − 43
TOP2A	5.54746	8.95599	5.97*E* − 44	3.91*E* − 42
COL2A1	10.5667	9.52236	1.00*E* − 43	6.53*E* − 42
APBA2	4.80478	5.88552	3.24*E* − 43	2.05*E* − 41
ARHGAP11A	4.48895	5.94823	3.83*E* − 43	2.42*E* − 41
ELOVL2	7.89597	5.7256	1.45*E* − 42	8.97*E* − 41
SIX2	12.5161	9.1566	1.20*E* − 41	7.12*E* − 40

Downregulated				
CLCNKA	−10.252	3.96865	2.05*E* − 202	3.78*E* − 198
MFSD4A	−6.5728	3.94457	1.29*E* − 200	1.19*E* − 196
SLC4A1	−8.7927	2.31542	2.03*E* − 196	1.25*E* − 192
AQP2	−13.939	7.07934	8.09*E* − 192	3.73*E* − 188
GCNT3	−7.5293	1.16448	6.64*E* − 186	2.45*E* − 182
PTGER1	−7.3274	2.50008	5.04*E* − 173	1.55*E* − 169
RASD1	−7.4598	5.22617	4.15*E* − 167	1.09*E* − 163
CLCNKB	−7.8428	3.94067	1.29*E* − 158	2.97*E* − 155
SLC4A9	−6.7956	-0.12053	2.48*E* − 153	5.08*E* − 150
BSND	−10.059	1.11375	4.06*E* − 151	7.49*E* − 148

mRNAs, messenger RNAs; FC, fold change; FDR, false discovery rate; CPM, counts per million.

**Table 2:** The top 10 differentially expressed miRNAs.

miRNA	log FC	log CPM	*P* value	FDR
Upregulated				
hsa-mir-483	6.21079	9.62843	4.15*E* − 13	1.38*E* − 11
hsa-mir-130b	4.81975	7.32181	3.45*E* − 12	9.49*E* − 11
hsa-mir-301b	6.27657	4.82704	7.19*E* − 12	1.89*E* − 10
hsa-mir-301a	3.12539	6.27469	3.30*E* − 11	7.11*E* − 10
hsa-mir-93	3.09801	14.43885	6.29*E* − 11	1.17*E* − 09
hsa-mir-342	2.501	7.7548	2.54*E* − 10	4.17*E* − 09
hsa-mir-218-1	3.6125	7.33389	2.57*E* − 10	4.17*E* − 09
hsa-mir-218-2	3.64285	7.30976	2.93*E* − 10	4.63*E* − 09
hsa-mir-149	3.16336	7.52028	8.65*E* − 09	1.22*E* − 07
hsa-mir-548f-1	6.76694	2.27932	9.05*E* − 09	1.24*E* − 07

Downregulated				
hsa-mir-934	−8.45898	-0.32026	1.29*E* − 111	8.18*E* − 109
hsa-mir-29c	−4.18422	7.45091	2.73*E* − 48	8.62*E* − 46
hsa-mir-203a	−5.55962	7.29738	1.15*E* − 31	2.43*E* − 29
hsa-mir-22	−3.04805	13.22851	2.54*E* − 31	4.01*E* − 29
hsa-mir-30a	−3.99689	15.05038	5.87*E* − 29	7.42*E* − 27
hsa-mir-29a	−4.0004	9.24513	1.46*E* − 28	1.54*E* − 26
hsa-mir-23a	−2.45158	8.87174	3.09*E* − 28	2.79*E* − 26
hsa-mir-29b-1	−3.36979	4.70744	9.67*E* − 26	7.64*E* − 24
hsa-mir-29b-2	−2.90603	5.11388	1.90*E* − 23	1.33*E* − 21
hsa-mir-27a	−2.49575	7.89127	3.41*E* − 22	2.15*E* − 20

miRNAs, microRNAs; FC, fold change; FDR, false discovery rate; CPM, counts per million.

**Table 3:** The top 10 differentially expressed lncRNAs.

lncRNA	log FC	log CPM	*P* value	FDR
*Upregulated*				
UG0898H09	9.4277	13.23352	1.15*E* − 27	9.76*E* − 26
AC099805.1	5.26719	8.90357	2.26*E* − 27	1.86*E* − 25
SLC16A1-AS1	3.20901	8.78361	1.65*E* − 26	1.28*E* − 24
SALRNA1	8.46703	6.97403	1.19*E* − 23	7.68*E* − 22
LINC02249	5.46785	6.42038	1.47*E* − 23	9.46*E* − 22
AL023803.2	5.42391	5.68452	4.23*E* − 22	2.55*E* − 20
AC108025.2	8.40171	6.28429	3.06*E* − 21	1.73*E* − 19
AC093702.1	11.75304	8.42543	3.58*E* − 21	2.01*E* − 19
IGF2-AS	7.04498	12.30149	6.89*E* − 21	3.76*E* − 19
AC079089.1	6.79084	7.90892	4.51*E* − 20	2.24*E* − 18

*Downregulated*				
AC093583.1	−10.96823	6.74312	1.46*E* − 249	1.49*E* − 245
AC124017.1	−8.36535	4.16573	7.57*E* − 160	3.87*E* − 156
AP005432.2	−7.03123	5.62924	2.31*E* − 115	7.88*E* − 112
LINC01543	−7.02138	3.42774	8.63*E* − 112	2.20*E* − 108
AL031710.1	−5.82991	5.00297	8.92*E* − 104	1.82*E* − 100
PP7080	−3.26355	10.33588	4.26*E* − 96	7.25*E* − 93
LINC00284	−8.75723	6.94171	1.39*E* − 94	2.03*E* − 91
LINC00671	−5.64455	3.84558	1.89*E* − 87	2.41*E* − 84
LINC01762	−6.23369	5.15653	3.74*E* − 87	4.25*E* − 84
AL049629.1	−6.8089	2.7119	3.27*E* − 82	3.34*E* − 79

lncRNAs, long noncoding RNAs; FC, fold change; FDR, false discovery rate; CPM, counts per million.

**Table 4:** Representative interactions between DELs and DEMis.

lncRNA	miRNA
IGF2-AS	hsa-mir-150, hsa-mir-17, hsa-mir-93
AC020907.1	hsa-mir-195, hsa-mir-497
HAS2-AS1	hsa-mir-150, hsa-mir-187
GDNF-AS1	hsa-mir-195, hsa-mir-497, hsa-mir-187
C2orf48	hsa-mir-93, hsa-mir-150, hsa-mir-195, hsa-mir-497, hsa-mir-17
	hsa-mir-183, hsa-mir-204
WT1-AS	hsa-mir-93, hsa-mir-96, hsa-mir-141, hsa-mir-200a
	hsa-mir-497, hsa-mir-17, hsa-mir-182, hsa-mir-429
	hsa-mir-25, hsa-mir-383, hsa-mir-195, hsa-mir-22
AC006305.1	hsa-mir-150, hsa-mir-195, hsa-mir-497, hsa-mir-17, hsa-mir-93
	hsa-mir-182, hsa-mir-204
	hsa-mir-25, hsa-mir-506, hsa-mir-383
LINC00284	hsa-mir-141, hsa-mir-200a, hsa-mir-195, hsa-mir-497
	hsa-mir-17, hsa-mir-508, hsa-mir-506, hsa-mir-93, hsa-mir-192
UCA1	hsa-mir-96, hsa-mir-182, hsa-mir-184, hsa-mir-506
	hsa-mir-383
AC061975.7	hsa-mir-93, hsa-mir-17
PSORS1C3	hsa-mir-551a, hsa-mir-301b, hsa-mir-454, hsa-mir-150
	hsa-mir-204
EGOT	hsa-mir-141, hsa-mir-200a, hsa-mir-195, hsa-mir-497
	hsa-mir-21, hsa-mir-183
COL18A1-AS1	hsa-mir-150, hsa-mir-187, hsa-mir-22
LINC00379	hsa-mir-93, hsa-mir-17

DELs, differentially expressed lncRNAs; DEMis, differentially expressed miRNAs; lncRNA, long noncoding RNA; miRNA, microRNA.

**Table 5:** The target genes of representative miRNAs in the ceRNA.

miRNA	mRNA
hsa-mir150	KLHL21, EGR2, MYB, SLC7A11
hsa-mir-93	CEP170, OSR1, ZNF280B, SMOC1, ATAD2, ENPP5, CHAF1A
	ITGB8, HMGB3, ARAP2, SAMD12, STAT3, ELAVL2, PARD6B
	ZBTB4, DENND5B, E2F2, IRF1, NACC2, POLQ, PRRG1, TET3, GINS4, NETO2, KIF23, FOXQ1
	ZBTB7A, E2F1, SYBU, GPR137C, RRM2, ANKRD33B, SACS
	EGR2, KIAA0922, KLF9, DUSP2, KPNA2, HAS2, FAM102A
hsa-mir-141	E2F3, H2AFZ, TIAM1, PTPRD, ELAVL4, MACC1, STAT4
	USP53, ELAVL2
hsa-mir-200a	USP53, PTPRD, H2AFZ, ELAVL2, MACC1, CCNE2
hsa-mir-182	PLEKHA8, CHL1, THBS1, MITF, FGF9, TCEAL7
hsa-mir-183	FAM217B, KIF2A, CCNB1, KLHL23
hsa-mir-429	PARD6B, ERMP1, DENND5B, TPD52L1, PRRG4, WASF3
hsa-mir-204	JARID2, SLC22A6, ARAP2, ARHGAP29, KLHL40, CDH2
	IL11, THRB, CHRDL1, HAS2
hsa-mir-383	IRF1
hsa-mir-506	SLC16A1, CD151, ZWINT
hsa-mir-192	ITGAV, TMPO, GRHL1
hsa-mir-454	IRF1 RBM20, JARID2, ZBTB4, CEP55, RACGAP1, MB21D2
	ENPP5, DEPDC1, SMOC1, EDN1, DLG5, CEP170
hsa-mir-22	TIAM1
hsa-mir-21	CPEB3, RASGRP1, TIMP3, CCL20, OSR1, PBRM1,
	TIAM1, SKP2, IL1B, STAT3, MSH2, ELOVL7

miRNAs, microRNAs; ceRNA, competitive endogenous RNA; mRNA, messenger RNA.

**Table 6:** The clinical feature data of WT patients.

Clinical feature	Variable	Patients *n* (%)
Age	≥1187	84 (70.00)
	<1187	36 (30.00)
Gender	Female	68 (56.67)
	Male	52 (43.33)
Race	White	89 (74.17)
	Black or African American	17 (14.17)
	Other	14 (11.67)
TNM stage	I	15 (12.50)
	II	48 (40.00)
	III	44 (36.67)
	IV	13 (10.83)

**Table 7:** Univariate analysis and multivariate analysis of OS for 120 patients.

Clinical feature	Univariate analysis			Multivariate analysis		
	*P* value	HR	95% CI	*P* value	HR	95% CI
Age	0.177	1.567	0.816 to 3.006	—		
Race	0.978	0.994	0.663 to 1.491	—		
Gender	0.032	0.539	0.307 to 0.947	0.005	0.432	0.242 to 0.773
TNM stage	0.015	1.497	1.083 to 2.070	0.002	1.674	1.203 to 2.329

## Data Availability

The data used to support the findings of this study are included within the article.
